# Tensor-Based Emotional Category Classification via Visual Attention-Based Heterogeneous CNN Feature Fusion

**DOI:** 10.3390/s20072146

**Published:** 2020-04-10

**Authors:** Yuya Moroto, Keisuke Maeda, Takahiro Ogawa, Miki Haseyama

**Affiliations:** 1 Graduate School of Information Science and Technology, Hokkaido University, N-14, W-9, Kita-ku, Sapporo, Hokkaido 060-0814, Japan; 2 Office of Institutional Research, Hokkaido University, N-8, W-5, Kita-ku, Sapporo, Hokkaido 060-0808, Japan; 3 Faculty of Information Science and Technology, Hokkaido University, N-14, W-9, Kita-ku, Sapporo, Hokkaido 060-0814, Japan; ogawa@lmd.ist.hokudai.ac.jp (T.O.); miki@ist.hokudai.ac.jp (M.H.)

**Keywords:** tensor analysis, visual attention, change with time, feature fusion, convolutional neural network

## Abstract

The paper proposes a method of visual attention-based emotion classification through eye gaze analysis. Concretely, tensor-based emotional category classification via visual attention-based heterogeneous convolutional neural network (CNN) feature fusion is proposed. Based on the relationship between human emotions and changes in visual attention with time, the proposed method performs new gaze-based image representation that is suitable for reflecting the characteristics of the changes in visual attention with time. Furthermore, since emotions evoked in humans are closely related to objects in images, our method uses a CNN model to obtain CNN features that can represent their characteristics. For improving the representation ability to the emotional categories, we extract multiple CNN features from our novel gaze-based image representation and enable their fusion by constructing a novel tensor consisting of these CNN features. Thus, this tensor construction realizes the visual attention-based heterogeneous CNN feature fusion. This is the main contribution of this paper. Finally, by applying logistic tensor regression with general tensor discriminant analysis to the newly constructed tensor, the emotional category classification becomes feasible. Since experimental results show that the proposed method enables the emotional category classification with the F1-measure of approximately 0.6, and about 10% improvement can be realized compared to comparative methods including state-of-the-art methods, the effectiveness of the proposed method is verified.

## 1. Introduction

Due to the increasing number of images on the Web, the demand for image understanding has increased [1,2,3]. Image understanding mainly focuses on two types of information: image-based information and human-based information. By using image-based information such as textures and luminance gradients, many researchers have tried to investigate semantic segmentation and object recognition [4,5,6,7,8,9]. Moreover, by using human-based information such as brain activities and gaze movements, many researchers have tried to investigate image emotion recognition and interest level estimation [10,11,12,13,14]. Therefore, we divide image understanding into image-based understanding and human-based understanding corresponding to the first and second types of information, respectively. Although the recent development of convolutional neural networks (CNNs) [4] has enabled the realization of image-based understanding with high performance [4,5,6,7,8,9], human-based understanding is still difficult since it is closely related to abstract semantics perceived by humans [15]. Image emotions lie on the highest level of abstract semantics, which can be defined as semantics describing the intensities and types of feelings, moods, affections, or sensibility evoked in humans viewing images [16]. In this study, we focus on the classification of images into emotional categories.

In studies on estimation of emotions evoked by humans gazing at images, the effectiveness of the use of several bio-signals has been mentioned [17,18,19,20]. It has been shown in the fields of psychology and neuroscience that human emotions are evoked by objects included in images [21,22]. Moreover, there is a relationship between emotional properties of an image and visual attention, i.e., the changes with time in visual attention are closely related to human emotions [23]. Therefore, in the same manner as emotion estimation, it is expected that the use of information on objects gazed at and information on changes in visual attention with time can be effective for emotional category classification.

In order to use the information on objects gazed at and information on changes in visual attention with time, we should obtain gaze data including the gazed locations of images and their duration times. Moreover, the objects in gazed areas need to be characterized by using CNNs which have been successfully implemented for object recognition. Therefore, the acquisition of gaze data and the training of CNNs for object recognition are needed for emotional category classification. Due to the burden of users to get a large amount of training gaze data, the number of images with gaze information is limited. On the other hand, CNNs need a large amount of training data. Thus, by using eye gaze data, the use of CNNs trained from scratch is not suitable for emotional category classification. It is necessary to use CNNs that are pre-trained by other domain datasets and extract outputs of an intermediate layer of the pre-trained CNN as CNN features. Extraction of CNN features is well known as one of transfer learning approaches [24]. In addition to consideration of objects that are gazed at, information on changes in visual attention with time is effective for emotional category classification as described above. Thus, this information should be dealt with together with consideration of objects gazed at. Then, in order to extract CNN features, we simply represent superimposed images and changes in visual attention with time. The superimposed representation is simple but effective for emotional category classification [25]. Therefore, for the collaborative use of CNN features and visual attention with changes over time, we treat the new image representation based on the superimposation and extract its CNN features.

Although CNN features have high representation ability for categories of the source domain, they do not necessarily have the ability for our target domain. Thus, for obtaining more semantic features and improving the representation ability to the image emotional category, it is desirable to use multiple CNN features calculated from multiple CNN models. Then, we have to consider the heterogeneous feature fusion method. For fusing heterogeneous CNN features, we should deal with not only changes over time but also interactions between CNN features. However, since CNN features have high dimensions, the fusion and analysis of the information are difficult. We therefore focus on tensor-based feature fusion like vector concatenation. The dimension of each mode of the constructed tensor is a lower dimension than that of vector concatenation. Thus, tensor-based feature fusion enables analysis of the changes over time and interactions between CNN features. However, we need to handle high-order information including information on CNN features themselves, the number of CNN features, and the changes over time. Consequently, for emotional category classification, a learning methodology with tensor analysis is strongly needed.

**Figure 1:**
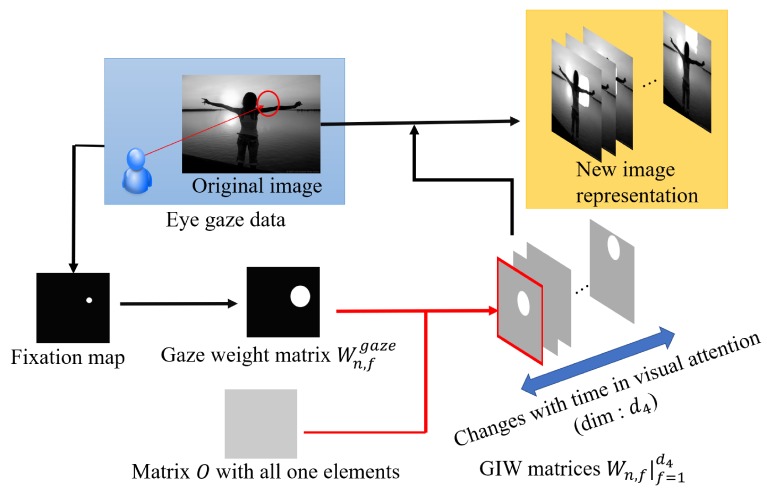
Overview of our new gaze-based image representation. Note that we handle color images in our method, but this figure shows a gray-scale version to visually explain our image representation. GIW matrices represent “gaze and image weight matrices”, which are explained in Section 3.1.

In this paper, we propose a new method for tensor-based emotional category classification via visual attention-based heterogeneous CNN feature fusion. In the proposed method, the new gaze-superimposed image representation [25] is adopted for associating images with eye gaze data as shown in Figure 1. Moreover, we extract multiple CNN features from each frame of the image representation. Note that the frame in the proposed method means the pair of the image and visual attention at each time unit that is divided the total gaze time in this image representation, although the term of frame is generally used for a movie. Furthermore, we extract several CNN features and construct a new CNN feature-based tensor (CFT) for considering the interactions of CNN features. Since each feature of the CFT is calculated from the gaze-based image representation, it can be expected that the proposed method will enable visual attention-based heterogeneous CNN feature fusion and that it will lead to improvement of the representation ability. Therefore, this CNN feature fusion based on a CFT is the main contribution of this paper. Finally, for the newly derived novel CFT, we perform supervised feature transformation based on general tensor discriminant analysis (GTDA) [26], which can transform original features into highly discriminant features, and realize emotional category classification based on logistic tensor regression (LTR) [27]. Consequently, accurate emotional category classification via the new feature fusion approach becomes feasible.

The rest of this paper is organized as follows. Related works and placement of this study are described in Section 2. In Section 3, tensor-based emotional category classification via visual attention-based heterogeneous CNN feature fusion is explained. The effectiveness of the proposed method verified from experimental results is shown in Section 4. In Section 5, we summarize this paper and present some discussions. Note that, in Appendix A, the mathematical notations, e.g., the tensor algebra, in this paper are shown.

## 2. Related Works

In this section, we introduce related works that focus on emotional category classification. Many researchers have focused on the dominant emotional category (DEC) when they classified images into emotional categories [28,29,30]. DEC means the emotional category that many humans evoke when they gaze at an image. There are several methodologies for tackling the DEC classification problem [28,31,32]. Furthermore, for constructing classifiers of emotional categories in these methods, image datasets with emotional categories have been published [29,30]. In [30], the dataset consists of images collected from Flickr, and the images are mainly realistic photos. Moreover, an abstract painting dataset for classifying different types of images closely related to emotional categories has been published [29]. In contrast to realistic photos, abstract paintings do not consist of clear objects and uniform colors. Thus, classification of such images by using only features directly calculated from the images is difficult.

Pasupa et al. proposed a classification method [28] using both eye gaze data, which are closely related to human emotions, and simple handcrafted visual features. On the other hand, since CNN features, which have more semantic information, have been used for visual features in recent years [24], Chen et al. proposed a CNN feature-based DEC classification method [32], and Rao et al. handled the outputs of some layers of a CNN trained from scratch [31]. In the DEC classification problem, it has been expected that CNN features are effective and that the collaborative use of eye gaze data and CNN features enables improvement in performance. Although these CNN-based methods certainly classify images into DECs with high performance, e.g., approximately 70% of classification accuracy [31,32], the large number of images which are pre-classified by humans for the training from scratch. Thus, CNN-based DEC classification methods are effective in the case that there exists the dataset with a large number of images already labeled emotional category [33], but images obtained from the domain which is different from the above dataset cannot be classified with high performance due to the lack of labeled data. Thus, in order to train CNN-based DEC classification methods for images obtained from the new domain, our method can help the label assignment problem since we can perform the training from the small number of training images. Therefore, human-based information such as gaze information is needed. In particular, since it is difficult to extract the emotion-related characteristics, gaze information is suitable for the DEC classification.

For improving the performance of the emotional category classification, it was reported in [10,34] that the use of multiple visual features is effective. Zhao et al. focused on the common factor between emotion features and each visual feature for predicting emotion distribution [10,34]. Based on the assumption that many images are pre-given to the emotion distribution, they extracted emotion features based on the emotion distribution from these images. Then, even though different visual features represent different semantics, they considered the relationships between emotion features and visual features but did not consider the relationships between visual features. Thus, although they used multiple visual features, their method cannot consider the interactions between visual features.

From the above discussion, we focus on the collaborative use of eye gaze data and multiple CNN features for image emotional category classification. In order to use multiple CNN features with consideration of their interactions, we newly introduce their feature fusion.

## 3. Tensor-Based Emotional Category Classification

**Figure 2:**
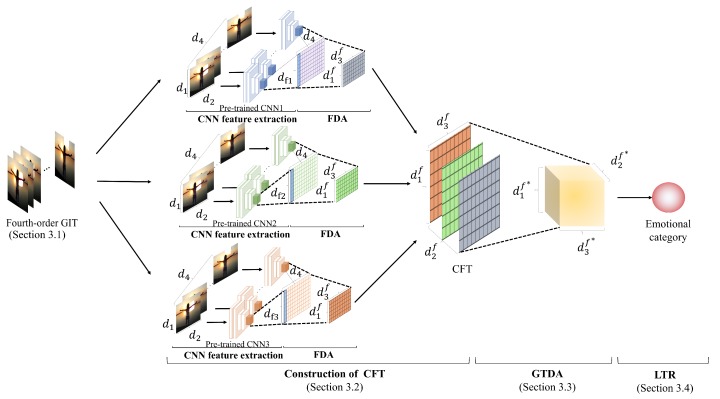
Overview of our method. We construct the new gaze-based image representation and extract multiple convolutional neural network (CNN) features. By aligning these CNN features, we construct a CNN feature-based tensor (CFT) and apply both general tensor discriminant analysis and logistic tensor regression to the CFT. Finally, our method classifies images into emotional categories using outputs of the proposed network. Details of the procedures are shown in Section 3.1, Section 3.2, Section 3.3 and Section 3.4.

In this section, we explain the proposed method. Our method classifies images into emotional categories via tensor-based analysis that enables realization of visual attention-based heterogeneous feature fusion suitable for our target problem. An overview of the proposed method is shown in Figure 2. Construction of the new gaze-based image representation for relating images and visual attention with changes over time is shown in Section 3.1. CNN feature extraction and construction of the CFT are shown in Section 3.2. Feature transformation based on GTDA and LTR-based emotional category classification using the transformed CFT are shown in Section 3.3 and Section 3.4, respectively. Since the effectiveness of the use of the combination of GTDA and LTR has been confirmed in [35], we adopt them in our method.

### 3.1. Construction of Gaze-Based Image Representation

In order to perform the new gaze-based image representation, the proposed method associates images with eye gaze data. We denote training images as $X_{n}^{\mathrm{image}}\in R^{d_{1}\times d_{2}\times d_{3}}$ (n=1,2,⋯,N; *N* being the number of training images). Note that the dimensions $d_{1}$, $d_{2}$, and $d_{3}$ correspond to the width and the height of an image and the number of color channels, i.e., three. In our method, a fixation map of each frame *f* ($=1,2,\cdots ,d_{4};d_{4}$ being the number of frames) is constructed on the basis of eye gaze data, and a Gaussian filter is applied to the obtained fixation map to obtain $W_{n,f}^{\mathrm{gaze}}\in R^{d_{1}\times d_{2}}$. Eye gaze data include data of gazed locations and their duration times, and we construct the fixation map by voting for pixel locations based on gazed locations. Then, a gaze and image weight (GIW) matrix $W_{n,f}\in R^{d_{1}\times d_{2}}$ of each frame *f* is calculated as follows:

(1) $$\begin{array}{c} W_{n,f}=d_{4}\frac{W_{n,f}^{\mathrm{gaze}}}{\sum_{f=1}^{d_{4}}W_{n,f}^{\mathrm{gaze}}}+O, \end{array}$$

where $O\in R^{d_{1}\times d_{2}}$ is a matrix for which the elements are all one. Finally, the image representation $X_{n}^{4\mathrm{th}}\in R^{d_{1}\times d_{2}\times d_{3}\times d_{4}}$ is calculated by using GIW as follows:

(2) $$\begin{array}{c} X_{n,col,f}^{4\mathrm{th}}=X_{n,col}^{\mathrm{image}}\circ W_{n,f}, \end{array}$$

where $X_{n,col,f}^{4\mathrm{th}}\in R^{d_{1}\times d_{2}}$ and $X_{n,col}^{\mathrm{image}}\in R^{d_{1}\times d_{2}}$ ($col=1,2,\cdots ,d_{3}$) are respectively obtained by matricizing $X_{n}^{4\mathrm{th}}$ and $X_{n}^{\mathrm{image}}$ for the mode of color channels. The operator “∘” means the calculation of the Hadamard product. The fourth-order GIT can reconstruct the original images as follows:

(3) $$\begin{array}{c} X_{n}^{\mathrm{image}}=\frac{1}{2d_{4}}\sum_{f=1}^{d_{4}}X_{n,f}^{4\mathrm{th}}. \end{array}$$

Thus, this representation consists of the image and the visual attention. By adopting this image representation, we extract CNN features with consideration of the changes in visual attention with time. In this way, construction of the new image representation, which is the input for the emotional category classification, becomes feasible.

### 3.2. Extraction of CNN Features and Construction of CFT

The proposed method extracts CNN features from the outputs of the last pooling layer of pre-trained CNNs. Specifically, we extract CNN features by using three kinds of state-of-the-art CNNs, DenseNet201 [36], InceptionResNet-v2 [37], and Xception [38]. It should be noted that the kinds and the number of CNNs are experimentally set in Section 4 since the purpose of this paper is to reveal the effectiveness of the use of multiple CNN features for the emotional category classification. Then, in this paper, we choose the above CNNs as the state-of-the-art methodologies. The dimensions of these CNN features are 1920, 1536, and 2048, respectively. In the proposed method, we construct the CFT by aligning these features. However, since the dimensions of these CNN features are different, their direct spatial concatenation is difficult. Thus, we apply supervised dimensionality reduction to these CNN features to unify their dimensions to the lowest one, i.e., 1536. In the proposed method, we simply adopt Fisher discriminant analysis (FDA) [39], which is one of the most well-known supervised dimensionality reduction methods. Finally, by aligning the CNN features, the proposed method constructs the CFT $V_{n}^{3\mathrm{rd}}\in R^{d_{1}^{f}\times d_{2}^{f}\times d_{3}^{f}}$. Note that $d_{1}^{f}$ means the minimum dimension of these CNN features and is equal to 1536, $d_{2}^{f}$ means the number of CNN features, i.e., three, and $d_{3}^{f}$ is the number of frames, $d_{3}^{f}=d_{4}$. The procedures shown in this subsection correspond to “construction of CFT” in Figure 2.

In the proposed method, we adopt the multiple CNN features for improving the representation ability. Moreover, our novel representation, CFT, can consider the dimensions of each CNN feature, changes in visual attention with time and kinds of CNN features. In this way, the proposed method simultaneously enables consideration of the interactions of multiple CNN features. Therefore, the proposed heterogeneous CNN feature fusion, i.e., the construction of the CFT, is expected to have high representation ability.

### 3.3. Feature Transformation Based on GTDA

We apply GTDA to $V_{n}^{3\mathrm{rd}}$ to obtain discriminative features that are suitable for emotional category classification. We define the class label $y_{n}\in \left\{0,1\right\}$ annotated to an image $X_{n}^{\mathrm{image}}$. Then, $y_{n}=1$ means that *n*-th image $X_{n}^{\mathrm{image}}$ includes a target label, i.e., a target emotional category. Note that, since each image has multiple emotional categories, the proposed method has to deal with multi-label problems, and the binary classification for each emotional category is thus adopted. In order to calculate the projection set ${\left\{P_{l}^{G^{*}}\in R^{d_{l}^{f}\times {d_{l}^{f}}^{*}}\right\}}_{l=1}^{3}$ (${d_{l}^{f}}^{*}<d_{l}^{f}$), we solve the following optimization problem:

(4) $$\begin{array}{c} P_{l}^{G^{*}}{|}_{l=1}^{3}=\arg\max_{P_{l}^{G}{|}_{l=1}^{3}}\mathrm{tr}\left({P_{l}^{G}}^{⊤}\left(S_{l}^{b}-\eta_{l}S_{l}^{w}\right)P_{l}^{G}\right), \end{array}$$

where $\eta_{l}$ is obtained as the largest eigenvalue of ${\left(S_{l}^{w}\right)}^{-1}S_{l}^{b}$ as shown in [26]. In addition, $S_{l}^{b}$ and $S_{l}^{w}$ are defined as follows:

(5) $$\begin{array}{c} S_{l}^{b}=\sum_{y\in \{0,1\}}\left[n_{y}{\mathrm{mat}}_{l}\left(\left(M_{y}-M\right)\times_{\bar{l}}{P_{l}^{G}}^{⊤}\right){\mathrm{mat}}_{l}^{⊤}\left(\left(M_{y}-M\right)\times_{\bar{l}}{P_{l}^{G}}^{⊤}\right)\right], \end{array}$$

(6) $$\begin{array}{c} S_{l}^{w}=\sum_{n=1}^{N}\left[{\mathrm{mat}}_{l}\left(\left(V_{n}^{3\mathrm{rd}}-M_{y_{n}}\right)\times_{\bar{l}}{P_{l}^{G}}^{⊤}\right){\mathrm{mat}}_{l}^{⊤}\left(\left(V_{n}^{3\mathrm{rd}}-M_{y_{n}}\right)\times_{\bar{l}}{P_{l}^{G}}^{⊤}\right)\right], \end{array}$$

where

(7) $$\begin{array}{c} M_{y}=\frac{1}{n_{y}}\sum_{n=1}^{N}(1-|y-y_{n}\left|\right)V_{n}^{3\mathrm{rd}}, \end{array}$$

(8) $$\begin{array}{c} M=\frac{1}{N}\sum_{y\in \{0,1\}}n_{y}M_{y}. \end{array}$$

Note that $M_{y}$ (y∈{0,1}) is the class mean tensor belonging to class *y*, and M is the total mean tensor of all training tensors. Note that $n_{y}$ is the number of images belonging to class *y*. Moreover, $M_{y}$ and M are all third-order tensors that lie in $R^{d_{1}^{f}\times d_{2}^{f}\times d_{3}^{f}}$. Finally, we obtain a tensor ${\hat{V}}_{n}^{3\mathrm{rd}}$ by transforming the CFT $V_{n}^{3\mathrm{rd}}$ as follows:

(9) $$\begin{array}{c} {\hat{V}}_{n}^{3\mathrm{rd}}=V_{n}^{3\mathrm{rd}}\prod_{l=1}^{3}\times_{l}P_{l}^{G^{*}}. \end{array}$$

Therefore, we can calculate highly discriminative features by using GTDA considering the categorical information.

### 3.4. Emotional Category Classification Based on LTR

In order to construct the LTR-based classifier, we use the transformed CFT ${\hat{V}}_{n}^{3\mathrm{rd}}$ as the input of LTR. Given ${\hat{V}}_{\mathrm{test}}^{3\mathrm{rd}}\in R^{{d_{1}^{f}}^{*}\times {d_{2}^{f}}^{*}\times {d_{3}^{f}}^{*}}$ from a test image, we try to estimate its class label $y_{\mathrm{test}}$. The LTR model used in the proposed method is formulated as follows:

(10) $$\begin{array}{c} \mathrm{Pr}\left[y_{\mathrm{test}}|{\hat{V}}_{\mathrm{test}}^{3\mathrm{rd}},Z\right]=\frac{1}{1+\exp(-\langle Z,{\hat{V}}_{\mathrm{test}}^{3\mathrm{rd}}\rangle )}, \end{array}$$

where Z, which is a parameter tensor of regression coefficients, is the same size as that of the transformed CFT ${\hat{V}}_{n}$. In order to obtain the optimal parameter tensor $\hat{Z}$ of Z, we solve the following maximum log-likelihood problem:

(11) $$\begin{array}{c} \hat{Z}=\arg\max_{Z}L(Z), \end{array}$$

where

(12) $$\begin{array}{cc} L\left(Z\right)=\sum_{n=1}^{N} & \left(y_{n}\ln\left(\langle Z,{\hat{V}}_{n}^{3\mathrm{rd}}\rangle \right)+\left(1-y_{n}\right)\ln\left(1-\langle Z,{\hat{V}}_{n}^{3\mathrm{rd}}\rangle \right)\right). \end{array}$$

We can solve the above maximization problem by adding $L_{1}$-norm regularization of Z based on the idea of [27].

Finally, the proposed method estimates the class label as follows:

(13) $$\begin{array}{c} y_{\mathrm{test}}=\arg\max_{y\in \{0,1\}}\mathrm{Pr}\left[y|{\hat{V}}_{\mathrm{test}}^{3\mathrm{rd}},\hat{Z}\right]. \end{array}$$

In this way, the proposed method realizes the heterogeneous CNN feature fusion and the tensor-based analysis with consideration of the changes in visual attention with time.

## 4. Experimental Results

We show experimental results in this section in order to verify the effectiveness of the proposed method. The experimental conditions are shown in Section 4.1 and the performance evaluation is shown in Section 4.2.

### 4.1. Experimental Conditions

A dataset of abstract paintings that contains 280 images [29] was used in the experiment. Each image was annotated by at least one emotion label(Images were rated by at least 14 persons in web-survey which was performed by Machajdik et al.) [29]. among eight emotional categories (*awe, amusement, contentment, anger, excitement, sad, disgust, and fear*). It should be noted that these emotional categories were defined by the psychological study on affective images [40]. We used these annotations as ground truths, and most of images have several emotion labels. Thus, we trained our method and comparative methods for each emotional category and each subject. From the 280 images, 224 images were randomly selected as training images and the remaining 56 images were used as test images to evaluate the performance of our emotional category classification method. For the evaluation measure, we adopted the F1-measure (F) obtained as follows:

(14) $$\begin{array}{c} F=\frac{2\times \mathrm{Recall}\times \mathrm{Precision}}{\mathrm{Recall}+\mathrm{Precision}}, \end{array}$$

where Recall and Precision are calculated by using the obtained classification results as follows:

(15) $$\begin{array}{c} \mathrm{Recall}=\frac{\mathrm{TP}}{\mathrm{TP}+\mathrm{FN}}, \end{array}$$

(16) $$\begin{array}{c} \mathrm{Precision}=\frac{\mathrm{TP}}{\mathrm{TP}+\mathrm{FP}}. \end{array}$$

TP, FN, and FP mean the numbers of images estimated to be true positive, false negative, and false positive, respectively. Since the number of images in the dataset was limited, evaluation was performed with a statistical test, Welch’s *t*-test [41], between our method and other methods, and the results are shown with the F1-measure.

Thirteen able-bodied subjects who were eleven healthy males and two healthy females, aged between 22 and 26 years (mean age: 23.5 ± 1.2 years) participated in the experiment. These subjects were normal or corrected-to-normal vision, and their eye gaze data were collected. The eye gaze data were obtained through Tobii Eye tracker 4C (https://tobiigaming.com/eye-tracker-4c/). Each subject gazed at images until evoking some emotions (This human research was conducted with the approval by the ethical committee in Hokkaido University.). These subjects just gazed at images, but we did not collect their evoked emotions, i.e., the ground truths were not their evoked emotions but labeled emotion labels provided by [29]. Their gaze was adjusted to the center of a display before showing a new image in one second. The gazing time length was normalized in such a way that it became $d_{4}$.

For comparison of the proposed method (PM), we adopted eight comparative methods as shown in Table 1. Comparative method 1 (CM1) does not use changes in visual attention with time. Therefore, in CM1, $d_{4}=1$ in the new image representation. Furthermore, CM1 uses only one CNN feature among three kinds of CNN features shown in Section 3.2. CM1 was adopted for evaluating the novel approaches introduced in this paper. We also adopted comparative method 2 (CM2), which uses only eye gaze features extracted on the basis of the state-of-the-art method [42], and we performed emotional category classification based on an extreme learning machine (ELM) [43]. CM2 was adopted for evaluating the use of the combination of image information and gaze information in our method. We also performed a comparison with the following three methods. We adopted a recently PM [28] that collaboratively uses eye gaze information and hand-crafted visual features as comparative method 3 (CM3). In the experiment, since multi-modal features such as gaze features and visual features were used, this fusion method is considered to be suitable for comparison. Qiu et al. proposed an emotional category classification method [44] by performing fusion of bio-information based on deep canonical correlation analysis (Deep CCA) [45]. Thus, we used the above state-of-the-art method as comparative method 4 (CM4) by using gaze features [42] and CNN features. Comparative method 5 (CM5) classifies images into emotional categories by applying feature fusion based on CCA [46] to both CNN features and gaze features [42]. Comparative method 6 (CM6) and comparative method 7 (CM7) use the CNN feature-fusion based on the vector concatenation. Concretely, CM6 makes the second-order CFT whose modes are the dimension of CNN features and the change over time. CM6 concatenates multiple CNN features at each time and applies GTDA to the constructed second-order CFT. Then, CM7 concatenates all of the CNN features, that is, CM7 treats the vector whose dimension is the dimension of CNN features times the kinds of CNN features. We took the average of the change with time in CNN features so as to prevent becoming a higher dimension. In order to handle the vector, CM7 applies FDA [39] instead of GTDA. CMs 6 and 7 classify these features into emotional categories based on a support vector machine (SVM) [47], which is one of the simplest classifiers, and ELM. Finally, comparative method 8 (CM8) fuses CNN features based on late fusion. In CM8, we first constructed a second-order CFT that consists of CNN features with consideration of the change over time for each CNN feature. Then, we applied GTDA and SVM or ELM to each second-order CFT and determined the final emotional category based on a softmax function, which is one of the simple but effective late fusion methods. Actually, in the use of multiple modalities, late fusion used in CM8 is applied [48,49].

### 4.2. Performance Evaluation

Table 2 and Table 3 show the results of the experiment. Table 2 shows the average of F1-measures of all emotional categories that were calculated for each subject. Table 3 shows those of all subjects that were calculated for each emotional category. “D”, “I”, and “X” represent DenseNet201, InceptionResNet-v2, and Xception, respectively. In our method, the combination order of CNN features influences the emotional category estimation performance by comparing PM (D-I-X), PM (D-X-I), and PM (X-D-I), and PM (D-I-X) outputs the best results on average. This is related to the mode expansion in the second mode of GTDA adopted in our method. PM (D-X-I), which has the worst results in PMs, outperforms all of the comparative methods. Thus, the effectiveness of PM is verified without considering the combination order of CNN features. The influence of this order has an interesting characteristic, and we should consider its decision method. However, in this paper, since we focus on heterogeneous CNN feature fusion and analysis, we will tackle this decision problem as our future work.

From the obtained results, the PM outperforms the comparative methods in the average of F1-measure. As shown in the results of PM with CM1, the effectiveness of the novel approach adopted in our method is verified. Moreover, the use of multiple CNN features is more effective for the emotional category classification than that using only one CNN feature. Then, it is expected that the greater the number of CNN features, the higher the accuracy of the emotional category classification. However, if the number of CNN features is four or more, the combination of CNN features increases. Thus, we simply used three CNN features for the simplicity in this experiment. The method which determines the optimal kinds and the number of CNN features should be investigated in the future work.

Comparing PM with CM1 and CM2 verifies that the new gaze-based image representation and CFT, i.e., the collaborative use of image and gaze information, are effective. Furthermore, since PM has a higher F1-measure than those of CM3 and CM4, which are recent and state-of-the-art frameworks, PM can classify images into emotional categories with high performance. A comparison of PM and CM5 indicates that the combination use of gaze information and images via both the new gaze-based image representation and CFT is more effective for emotional category classification than the baseline fusion method. Moreover, a comparison of PM with CMs 6, 7, and 8 shows that the proposed heterogeneous CNN feature fusion and its analysis are more effective than the vector-based concatenation methods for emotional category classification. Then, the tendency of experimental results of CM8 is different from that of other methods according to the difference of the fusion method [50]. Concretely, CM8 adopted the late-fusion method which generally provides high performance when the performance of each method to be fused is close.

We also show the results of Welch’s *t*-test between PM (D-I-X) and the comparative methods. Since the *p*-value is lower than 0.05, PM is statistically superior to CMs 1–5 and 7. On the other hand, the results for CMs 6 and 8 have higher *p*-values than those of other comparative methods since CMs 6 and 8 utilize the change in CNN features with time in the same manner as that in PM. The differences between PM, CM 6, and CM 8 are only the concatenation method and when to concatenate heterogeneous modalities. In other words, CMs 6 and 8 are similar to PM. However, these differences are considered to cause the slight improvement of the classification performance.

**Figure 3:**
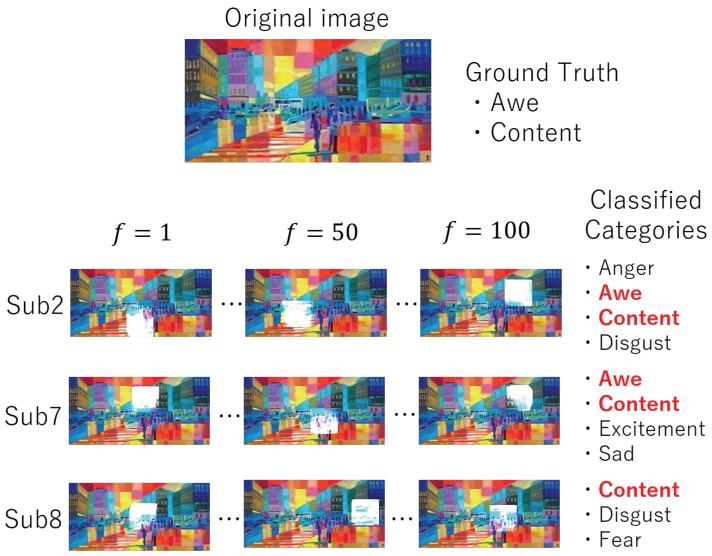
This figure shows some experimental results of some test images and their ground truths. The areas that the subjects gazed at are shown in white at frames 1, 50, and 100. From these gaze data, PM (D-I-X) classifies this image into some categories. If the classified category is the same as the ground truth, the corresponding category is indicated in red. If the classified category is false, the corresponding category is indicated in black.

In addition to the quantitative evaluations, we show one of the experimental results in Figure 3. In Figure 3, if the classified category is the same as the ground truth, the corresponding category is indicated in red. If the classified category is false, the corresponding category is indicated in black. Although the gaze-based image representation of Subs 2 and 7 are classified into four categories including all of the GTs that of Sub 8 is classified into three categories including one of the GTs. Concretely, although Subs 2 and 7 gazed at almost the same area at each frame in the image shown, Sub 8 gazed at a different area. This difference causes the difference in classified emotional categories. Thus, we confirmed that the change in visual attention with time is related to human emotions.

## 5. Discussion and Conclusions

In this paper, we presented an emotional category classification method based on tensor analysis that realizes the visual attention-based heterogeneous CNN feature fusion. In order to improve the classification performance, the PM constructs the new tensor, CFT, that integrates the outputs from the multiple CNN architectures with consideration of the changes in visual attention with time. Consequently, emotional category classification becomes feasible by using GTDA and LTR. Experimental results verified the effectiveness of the PM. In the experiment, we used only one image dataset in consideration of the burden on the subjects. Obtaining eye gaze data is a great burden for the subjects. Since such a task may prevent verifying the correct effectiveness, we used only one dataset. However, the use of an abstract painting dataset is more suitable than realistic image datasets for emotional category classification. Thus, there is no lack of the effectiveness of the PM with respect to the number of images in this dataset. In a future work, we will use other datasets in order to verify the robustness of our method.

## Figures and Tables

**Table 1:** The difference of the proposed method (PM) and comparative methods (CMs). The marks ‘√’ and ‘X’ mean that the corresponding method considers or does not consider the time change. Moreover, “Softmax” means that we applied the softmax function to the outputs of several classifiers and obtained probabilities. Then, classification was performed based on the value obtained by multiplying these probabilities. Furthermore, “Hand-crafted feature” means that CM3 extracted hand-crafted visual features such as Gabor filter-based and Sobel filter-based visual features from images obtained by superimposing original images and fixation maps.

	Time Change	Gaze Feature	Fusion
PM	√	GIT	CFT
CM1	X	GIT	CFT
CM2	X	Novel gaze feature [42]	Only gaze feature
CM3	X	Hand-crafted feature [28]	Concatenation
CM4	X	Novel gaze feature [42]	DeepCCA [45]
CM5	X	Novel gaze feature [42]	CCA [46]
CM6	√	GIT	Concatenation
CM7	X	GIT	Concatenation
CM8	√	GIT	Softmax

**Table 2:** Average values of F1-measures of all emotional categories calculated for each subject. Note that •-•-• and •,•,• are different in terms of whether their order is considered or not. *p*-value was obtained by Welch’s *t*-test [41].

	PM	PM	PM	CM1	CM1	CM1	CM2 [42]	CM3 [28]	CM4 [44]	CM5 [46]	CM6	CM6	CM7	CM7	CM8	CM8
CNN Feature	D-I-X	D-X-I	X-D-I	D	I	X	-	-	D,I,X	D,I,X	D,I,X	D,I,X	D,I,X	D,I,X	D,I,X	D,I,X
Classifier	ELM	ELM	ELM	ELM	ELM	ELM	ELM	SVM	SVM	SVM	SVM	ELM	SVM	ELM	SVM	ELM
Sub1	0.616	0.613	0.631	0.426	0.468	0.452	0.567	0.546	0.512	0.529	0.533	0.543	0.578	0.544	0.531	0.560
Sub2	0.616	0.591	0.635	0.514	0.429	0.486	0.402	0.371	0.311	0.455	0.508	0.519	0.413	0.414	0.506	0.570
Sub3	0.592	0.552	0.538	0.469	0.415	0.362	0.490	0.379	0.567	0.538	0.531	0.567	0.442	0.501	0.494	0.498
Sub4	0.567	0.558	0.583	0.401	0.417	0.423	0.402	0.520	0.532	0.540	0.502	0.563	0.503	0.571	0.468	0.527
Sub5	0.606	0.603	0.551	0.507	0.446	0.441	0.512	0.488	0.564	0.502	0.489	0.560	0.504	0.525	0.473	0.528
Sub6	0.603	0.542	0.589	0.486	0.453	0.440	0.505	0.397	0.478	0.512	0.495	0.526	0.504	0.498	0.446	0.528
Sub7	0.565	0.643	0.593	0.393	0.498	0.341	0.521	0.396	0.440	0.489	0.487	0.592	0.513	0.507	0.469	0.546
Sub8	0.567	0.597	0.598	0.378	0.424	0.533	0.468	0.473	0.567	0.537	0.510	0.511	0.565	0.588	0.400	0.515
Sub9	0.598	0.558	0.597	0.471	0.436	0.414	0.505	0.463	0.565	0.479	0.503	0.475	0.498	0.433	0.484	0.539
Sub10	0.560	0.526	0.603	0.463	0.395	0.400	0.425	0.471	0.522	0.563	0.487	0.483	0.447	0.474	0.451	0.555
Sub11	0.628	0.564	0.568	0.465	0.431	0.433	0.423	0.468	0.468	0.494	0.548	0.521	0.497	0.492	0.408	0.599
Sub12	0.597	0.597	0.588	0.333	0.442	0.408	0.414	0.406	0.497	0.466	0.509	0.515	0.562	0.529	0.489	0.625
Sub13	0.570	0.579	0.598	0.503	0.487	0.381	0.667	0.537	0.409	0.495	0.550	0.537	0.436	0.440	0.506	0.584
Average	0.591	0.579	0.590	0.447	0.442	0.424	0.485	0.453	0.487	0.502	0.512	0.532	0.497	0.503	0.471	0.552
*p*-value				(*p* < 0.01)	(*p* < 0.01)	(*p* < 0.01)	(*p* < 0.01)	(*p* < 0.01)	(*p* < 0.01)	(*p* < 0.01)	(*p* < 0.01)	(*p* < 0.01)	(*p* < 0.01)	(*p* < 0.01)	(*p* < 0.01)	(*p* < 0.01)

**Table 3:** Average values of F1-measures of all subjects calculated for each emotional category. Note that •-•-• and •,•,• are different in terms of whether their order is considered or not. *p*-value was obtained by Welch’s *t*-test [41].

	PM	PM	PM	CM1	CM1	CM1	CM2 [42]	CM3 [28]	CM4 [44]	CM5 [46]	CM6	CM6	CM7	CM7	CM8	CM8
CNN Feature	D-I-X	D-X-I	X-D-I	D	I	X	-	-	D,I,X	D,I,X	D,I,X	D,I,X	D,I,X	D,I,X	D,I,X	D,I,X
Classifier	ELM	ELM	ELM	ELM	ELM	ELM	ELM	SVM	SVM	SVM	SVM	ELM	SVM	ELM	SVM	ELM
Amusement	0.667	0.667	0.667	0.409	0.473	0.564	0.527	0.418	0.531	0.501	0.486	0.648	0.488	0.489	0.462	0.622
Anger	0.667	0.667	0.667	0.605	0.506	0.446	0.527	0.449	0.493	0.517	0.502	0.493	0.505	0.545	0.483	0.490
Awe	0.667	0.667	0.667	0.360	0.354	0.410	0.529	0.453	0.469	0.536	0.387	0.500	0.648	0.457	0.540	0.526
Content	0.424	0.414	0.394	0.426	0.443	0.355	0.411	0.498	0.497	0.503	0.553	0.501	0.502	0.514	0.389	0.519
Disgust	0.667	0.667	0.667	0.452	0.481	0.475	0.521	0.435	0.505	0.476	0.420	0.488	0.460	0.450	0.524	0.481
Excitement	0.550	0.599	0.630	0.431	0.450	0.419	0.434	0.494	0.475	0.500	0.527	0.533	0.480	0.482	0.544	0.622
Fear	0.612	0.523	0.563	0.452	0.388	0.366	0.531	0.441	0.456	0.495	0.660	0.547	0.530	0.551	0.406	0.551
Sad	0.474	0.426	0.468	0.438	0.439	0.358	0.402	0.435	0.473	0.487	0.558	0.544	0.543	0.533	0.421	0.602
Average	0.591	0.579	0.590	0.447	0.442	0.424	0.485	0.453	0.487	0.502	0.512	0.532	0.497	0.503	0.471	0.552
*p*-value				(*p* < 0.01)	(*p* < 0.01)	(*p* < 0.01)	(*p* < 0.05)	(*p* < 0.01)	(*p* < 0.05)	(*p* < 0.05)	(*p* < 0.06)	(*p* < 0.08)	(*p* < 0.02)	(*p* < 0.05)	(*p* < 0.01)	(*p* < 0.2)

## Appendix A. Mathematical Notations

The order of a tensor corresponds to the number of modes. In this paper, each lowercase letter, e.g., *a*, represents a scalar, each boldface capital letter, e.g., A, represents a matrix (second-order tensor) and each calligraphic letter, e.g., A represents a tensor (third-order tensor or higher tensor).

The mode-*l* matricizing of a *k*th-order tensor $A^{k\mathrm{th}}\in R^{{}^{D_{1}\times D_{2}\times \cdots D_{k}}}$ is denoted by mat${}_{l}\left(A^{k\mathrm{th}}\right)\in R^{{}^{D_{l}\times \prod_{i\neq l}D_{i}}}$, which is the ensemble of vectors $\in R^{m_{l}}$ obtained by keeping the *l*th mode fixed and varying the other modes. The mode-*l* product of a *k*th-order tensor $A^{k\mathrm{th}}$ is denoted as $A^{k\mathrm{th}}\times_{l}B_{l}\in R^{{}^{D_{1}\times D_{2}\times \cdots \times D_{l-1}\times D_{l}^{*}\times D_{l+1}\times \cdots \times D_{k}}}$ by using a matrix $B_{l}\in R^{{}^{D_{l}\times D_{l}^{*}}}$. Several multiplications are described as follows:

(A1) $$\begin{array}{c} A^{k\mathrm{th}}\times_{\bar{l}}B_{l}=A^{k\mathrm{th}}\times_{1}B_{1}\times_{2}B_{2}\times \cdots \times_{l-1}B_{l-1}\times_{l+1}B_{l+1}\times \cdots \times_{k}B_{k}. \end{array}$$

The inner product is denoted by 〈A,B〉, where the size of B is the same as that of A. The above notations are based on those used in previous reports [26,35].
